# Induction of Immunological Tolerance by Oral Anti-CD3

**DOI:** 10.1155/2012/425021

**Published:** 2011-11-14

**Authors:** Andre Pires da Cunha, Howard L. Weiner

**Affiliations:** Center for Neurologic Diseases, Brigham and Women's Hospital, Harvard Medical School, Boston, MA 02115, USA

## Abstract

In recent years, our knowledge about immunoregulation and autoimmunity has significantly advanced, but nontoxic and more effective treatments for different inflammatory and autoimmune diseases are still lacking. Oral tolerance is of unique immunologic importance because it is a continuous natural immunologic event driven by exogenous antigen and is an attractive approach for treatment of these conditions. Parenteral administration of anti-CD3 monoclonal antibody is an approved therapy for transplantation in humans and is effective in autoimmune diabetes. Orally administered anti-CD3 monoclonal antibody is biologically active in the gut and suppresses experimental models of autoimmune diseases. Orally delivered antibody does not have side effects including cytokine release syndromes, thus oral anti-CD3 antibody is clinically applicable for chronic therapy. Here we review findings that identify a novel and powerful immunologic approach that is widely applicable for the treatment of human autoimmune conditions.

## 1. Introduction

Understanding how the immune system balances between tolerance and protective immunity is still a key challenge in immunology. Although several approaches have been used to treat autoimmune diseases, they usually involve nonspecific immunosuppression, which frequently comes along with several undesirable side effects. Thus, one of the major goals for the immunotherapy of these pathologies is the induction of regulatory T cells that mediate immunologic tolerance. In this scenario, the gut environment is particularly important, as tolerance induction is the default immune pathway at this site in physiological conditions [1]. The immunological tolerance to antigens that gain access to the body via the oral route has been termed “oral tolerance” [2] and it has been classically defined as the specific suppression of cellular and/or humoral immune responses to an antigen that was first administered by the oral route [1, 3, 4]. 

Studies of oral tolerance have classically involved the administration of oral antigen followed by challenge with the homologous antigen (usually in an adjuvant) to demonstrate antigen-specific tolerance. In this context, an experimental system that has been frequently used for the study of T-cell function in oral tolerance is the use of TCR transgenic (Tg) mice in which all T cells have a common TCR. Using TCR Tg mice, we administered the cognate antigens myelin basic protein (MBP) and ovalbumin (OVA) and investigated how oral administration of an antigen-affected specific T-cell subsets. In these studies, we demonstrated the dose-dependent induction of Tregs in MBP TCR Tg mice [5] and deletion following high-dose oral administration of OVA in OVA TCR Tg mice [6]. 

During the course of our experiments, we found that feeding OVA to OVA TCR Tg mice induced CD4+CD25+ Treg cells [7, 8]. Other investigators also showed that oral antigen induced CD4+CD25+ Tregs [9]. The CD4+ cells from OVA TCR Tg fed animals had greater suppressive properties *in vitro* than natural Tregs, mediated suppression in part by both TGF-*β* and IL-10, and presented increased expression of CTLA-4, a molecule known to be involved in Treg activity [6, 10]. Although, these findings demonstrated that oral antigen could induce/expand Tregs, administration of OVA to OVA TCR Tg mice is dependent on TCR Tg mice and not translatable to humans. We thus asked whether it was possible to trigger the TCR in the gut CD4+ T cells of wild-type mice and induce Tregs without using cognate antigen. This possibility will be discussed in more detail below.

## 2. Immune Tolerance and Anti-CD3 Antibody Treatment

Different mechanisms have been implicated in the induction and maintenance of immune tolerance including deletion, anergy, and active cellular regulation [11]. One approach that has been successfully used for the induction/restoration of immune tolerance is the administration of CD3-specific antibody. Different groups have demonstrated that parenteral administration of anti-CD3 is effective not only in animal models of autoimmunity, including autoimmune diabetes [12–14] and experimental autoimmune encephalomyelitis (EAE) [15, 16] but also in human trials of autoimmune diabetes [17–19] and psoriatic arthritis [20]. Furthermore, intravenous administration of anti-CD3 is an approved therapy for acute transplant rejection in humans although side effects related to cytokines release limit its chronic use [21, 22]. Humanized antibodies have been designed to reduce these side effects [23, 24] but the successful translation might require new therapies that would be more physiologic and less toxic and mucosal tolerance can be exploited in this direction.

### 2.1. Oral Anti-CD3

It is known that anti-CD3 binds to the *ε* chain of the TCR complex and when given intravenously deletes T cells and, as mentioned above, has been shown to be an effective treatment for type 1 diabetes in the non-obese diabetic (NOD) mouse [25]. We hypothesized that oral administration of anti-CD3 monoclonal antibody would replace the use of a cognate antigen to trigger the TCR and would thus induce Tregs when given orally. Monoclonal antibodies have not been given orally on the assumption that they would be degraded in the gut and thus would not be biologically active. Nonetheless, it is known that orally administered cytokines [26] and peptides [27, 28] are biologically active, demonstrating that orally administered proteins are not completely degraded in the gut. 

Thus, to test this hypothesis, we administered hamster anti-mouse CD3 (2C11 clone) to SJL mice and immunized with PLP/CFA to induce EAE. We found that oral anti-CD3 suppressed both clinical and pathologic features of EAE both in the PLP and MOG EAE model [29]. There was a dose effect observed, with EAE suppression by oral anti-CD3 seen at lower (5 *μ*g), but not higher doses (50 *μ*g, 500 *μ*g). These findings were consistent with the classic paradigm of oral tolerance in which induction of Tregs is seen at lower but not higher doses [1]. Furthermore, it demonstrates that induction of Tregs by oral anti-CD3 is not simply related to administering large amounts of antibody to overcome degradation of the antibody in the gut. Indeed, biologically active anti-CD3 could be isolated from intestinal eluates of animals that were given anti-CD3 orally [30] and we could also visualize anti-CD3 being taken up by gut epithelial cells and binding to gut DCs in intestinal loop experiments [1]. The extent to which these DCs that take up anti-CD3 play a role in the mechanism of action of oral anti-CD3 has still to be better studied. Of note, the Fc portion of anti-CD3 was not required, as anti-CD3 Fab′2 fragment is active orally and induces Tregs.

### 2.2. Differences between Intravenous and Oral Anti-CD3 Antibody

It is known that intravenous anti-CD3 enters the blood stream, modulates CD3 from the cell surface, and leads to the depletion of CD3+ T cells. Oral anti-CD3, on the other hand, does not enter the blood stream or modulate CD3 from the cell surface but acts locally in the gut to induce Th3 type CD4+CD25−LAP+ Tregs in the MLNs. As oral anti-CD3 does not enter the bloodstream, there is no cytokine release syndrome, one of the main problems with the intravenous administration of anti-CD3. In the EAE model, intravenous anti-CD3 is effective when given after disease manifests but not when given prior to disease induction. Oral anti-CD3, on the other hand, ameliorates EAE both when given prior to EAE induction and at the height of disease [29]. The explanation for this difference is related to the fact that intravenous anti-CD3 acts primarily by lysing disease effector cells (present only after disease induction), whereas oral anti-CD3 acts by inducing Tregs. Intravenous anti-CD3 has been reported to induce Tregs that act in a TGF-*β*-dependent fashion, but only after lysis of T cells [12]. Therefore, immunoregulation by oral anti-CD3 involves different mechanisms other than intravenous anti-CD3 and has the advantage of being safer as it is not associated with systemic side effects [31].

We found that oral anti-CD3 ameliorates disease in other autoimmune and inflammatory diseases including streptozocin-induced [30] and NOD autoimmune diabetes (unpublished data), type 2 diabetes in the Ob/Ob mouse [32], lupus prone SNF1mice [33], and collagen-induced arthritis [34]. A different group of investigators reported that oral anti-CD3 suppresses atherosclerosis in ApoE^−/−^ mice [35]. In all these models, disease amelioration was related to the induction of TGF-*β*-dependent Tregs that express LAP on their surface. Indeed, *in vivo* treatment of animals with anti-LAP antibody [36], abrogated tolerance induction by oral anti-CD3 in the EAE model (da Cunha et al. [1], unpublished). We also found that nasal anti-CD3 ameliorates lupus but does so by inducing an IL-10-dependent CD4+CD25−LAP+ Treg as opposed to the TGF-*β*-dependent LAP+ Treg induced by oral anti-CD3 [37]. This is consistent with the observation that GALT DCs induce TGF-*β*-dependent Tregs versus IL-10-dependent Tregs induced in the bronchial associated lymphoid tissue [38]. Furthermore, these results demonstrate that mucosally administered anti-CD3 appears to act in a fashion analogous to mucosally administered cognate antigen [38]. 

The effects of oral anti-CD3 raise the question whether it is more advantageous to induce antigen-specific versus antigen nonspecific Tregs for the treatment of autoimmune and inflammatory diseases. It is assumed that the induction of antigen-specific Tregs is preferable, as one would have specific immune modulation with less potential side effects. Furthermore, because of the phenomenon of bystander suppression, which we first described in association with oral tolerance [39], cytokines such as TGF-*β* released from antigen-specific Tregs at the target organ would suppress reactivity to other autoantigens that developed in the course of epitope spreading. Of note, there may be target organ specificity even when antigen nonspecific Tregs are induced with oral anti-CD3 as we observed increased numbers of Th3 type LAP+ Tregs in the pancreatic lymph nodes of autoimmune diabetic mice [30], and this has been suggested for atherosclerosis models [29]. Furthermore, in conditions such as type 2 diabetes, lupus, and atherosclerosis, there are not well-defined target antigens and in these conditions induction of antigen nonspecific Tregs by anti-CD3 may be preferable. Studies are in progress to test the combination of mucosal anti-CD3 given with antigen. As discussed below, oral anti-CD3 is currently being tested in humans.

## 3. Oral Anti-CD3 in Humans

Oral anti-CD3 has been tested in a phase 1 study in healthy human volunteers (3 per group) who were orally administered 0.2, 1.0, or 5.0 mg of mouse anti-human OKT3 mAb daily for 5 days [31]. Immunologic effects were observed in the peripheral blood and consisted of transient proliferation, suppression of Th1/Th17 responses, increased expression of Treg markers, increased TGF-*β*/IL-10, and decreased IL-23/IL-6 expression in dendritic cells. No side effects were observed. There were no human anti-mouse antibody responses, changes in CD3 cells in the blood, or modulation of CD3 from the surface of T cells. The optimal dose was found to be 1 mg. 

Oral OKT3 mAb has also been recently tested in a single-blind randomized placebo-controlled phase 2a study in patients with nonalcoholic steatohepatitis (NASH) and altered glucose metabolism that included subjects with type-2 diabetes [40]. The study was performed at the Hadassah-Hebrew University Medical Center in Jerusalem, Israel. OKT3 or placebo was orally administered (9 per group) at doses of 0.2, 1.0, and 5.0 mg. In the NASH study 36 subjects were treated once daily for 30 days with final follow-up 60 days after the first dose.

Oral OKT3 was safe with no adverse effects or systemic toxicity as measured by blood hematology, chemistry, immunological safety markers, and physical signs. There were no changes in blood levels of CD3, CD4, or CD8-positive cells. Oral OKT3 induced regulatory T cells, which generally persisted to day 60 and trends in cytokine production consistent with effects observed in the phase 1 clinical study and in animal models. Positive trends in clinical parameters, some of which were statistically significant were also observed including a reduction in liver enzymes and reduced blood levels of glucose and insulin. Several of the positive efficacy trends persisted to day 60 following cessation of treatment at day 30. Some subjects had increased levels of serum antibodies directed against OKT3, which did not affect the positive trial results observed. These results suggest that oral anti-CD3 mAb may have clinical benefit for subjects with NASH or type-2 diabetes. Confirmatory studies are now needed, including studies with humanized antibodies. These results will provide the basis for investigating oral/nasal anti-CD3 in other autoimmune and inflammatory conditions in humans.

Recently, an important development that will facilitate studies related to the use of humanized anti-CD3 antibodies was the generation of transgenic mice expressing the *ε* chain of the human CD3 complex on the NOD background (NOD-huCD3*ε*). These mice develop spontaneous diabetes to the same extent as the wild-type NOD mice and when treated with a humanized anti-CD3 antibody, they exhibited a complete and durable disease remission [41]. This model will be very useful for studies evaluating anti-human CD3 antibodies, including its administration by oral route, and this will open new perspectives and clarify their potential clinical utility.

## 4. Summary

Mucosal tolerance is an attractive approach for the treatment of autoimmune inflammatory diseases as it is a clinically applicable physiologic manner to suppress inflammation through the induction of regulatory T cells. Its lack of toxicity and ease of administration are also important features desired for an effective immunotherapy. Several studies have demonstrated that oral (or nasal) administration of anti-CD3 monoclonal antibodies can be used to induce immune tolerance and is effective in the treatment of animal models of autoimmune diseases. These studies have also demonstrated that mucosal administration of anti-CD3 antibodies does not lead to detectable side effects including cytokine release syndrome or antiglobulin response and thus could be used as a continuous therapy for the treatment of these conditions. In summary, results presented here indicate that oral administration of anti-CD3 antibodies represents a new avenue that can be investigated for the treatment of autoimmune diseases.
